# Multidimensional Performance Assessments in U15 Female Soccer: The Predictive Validity for Different Selection Levels in U17 and Success in Adulthood

**DOI:** 10.1002/ejsc.12335

**Published:** 2025-06-26

**Authors:** Daniel Leyhr, Fynn Bergmann, Johannes Raabe, Oliver Höner

**Affiliations:** ^1^ Institute of Sports Science Eberhard Karls University of Tübingen Tübingen Germany; ^2^ Methods Center Eberhard Karls University of Tübingen Tübingen Germany; ^3^ University of North Georgia Dahlonega Georgia USA

**Keywords:** football, prognostic relevance, skills, talent diagnostics, talent identification and development

## Abstract

Female players have largely been neglected in soccer‐specific talent research. Particularly, prospective studies examining the predictive validity of multidimensional performance factors for different selection levels—especially players' success in adulthood—remain scarce. This prospective cohort study investigated the predictive validity of multidimensional performance factors for the future success of U15 female players (*N* = 264) participating in the German Football Association's Talent Development program. All players' kicking, tactical, and psychosocial skills, as well as endurance capacity, were evaluated subjectively. They also completed five objective motor tests (sprint, agility [CODS], dribbling, ball control, and juggling). Players' future success was based on their selection for the U17 Bundesliga (43.6% selection rate), U17 Youth National Team (6.4%), and Women's Bundesliga (i.e., professional level in adulthood; 6.1%). Univariate analyses examined performance differences in each predictor between selected and nonselected players. Multivariate analyses determined whether objective predictors, subjective predictors, or a combination thereof offered the best prediction. Univariate results indicate the predictive validity of both assessments, revealing a trend toward larger effect sizes for higher selection levels and differences in predictor relevance. Multivariate models showed that adding subjective to objective predictors led to an increase in explained variance for participation in the U17 Bundesliga (Nagelkerke's *R* ^2^ = 16%) and Women's Bundesliga (31%), but not U17 Youth National Team (18%). Sprint performance reached significance across models, whereas the sole significant subjective predictor was tactical skills regarding participation in the Women's Bundesliga. Overall, these findings support the practical value of both assessments, which can likely be increased further via more distinct coach ratings.

**Summary**

- The findings support the consideration of multidimensional performance factors in the context of talent identification and development in elite female soccer.
- The combination of subjective and objective assessments can enhance the predictive power regarding U15 players' future success in late adolescence and adulthood.
- Although objective assessments were particularly suitable for evaluating distinct (motor) predictors, the contribution of subjective coach ratings may be improved through more nuanced evaluations of theoretically distinct competence areas.

## 1 Introduction

Talent identification and development (TID) research is a continuously growing area of scholarship with investigations of multifaceted topics across a variety of sports (Baker et al. 2020; Barraclough et al. 2022). Yet, one meaningful limitation of the existing empirical evidence is the underrepresentation of female athletes in study samples—a disparity referred to as the gender data gap in talent development (Curran et al. 2019). TID research in soccer is no exception to this trend (Murr et al. 2018; Murr, Raabe, et al., 2018; Okholm Kryger et al. 2021; Williams et al. 2020; Wrang et al. 2021), which is surprising given not only the worldwide increase in participation rates among girls and women but also the popularity of professional women's soccer tournaments over recent decades (Dunn 2016; Fédération Internationale de Football Association 2021).

To further support this ongoing rise in participation, popularity, and professionalization, Fédération Internationale de Football Association (2018) launched the “Women's Football Strategy” and encouraged all national football associations to enhance developmental programs for female soccer, including those aimed at optimizing TID (e.g., empowering girls to get involved, modernizing infrastructures, and increasing the number of qualified female coaches). Although improvements are reflected in a 24% increase in the number of active women and girls playing football since 2019 (i.e., 16.6 million in 2023; FIFA, 2023a, p. 8), implementation of elite talent pathways, and reformed competitive league systems, these processes are far from being completed (see also FIFA, 2023b).

That said, research exploring the identification and development of talent in female youth players can help refine such processes and, in turn, advance the professionalization of women's soccer. A crucial factor in supporting practitioners in TID processes is the availability of empirical insights into performance factors that reveal predictive power for players' future success (Höner et al. 2023). Based on a systematic review of prospective studies, Williams et al. (2020) proposed a multidimensional model of talent predictors in soccer, encompassing physical, skill‐related, psychological, and sociological characteristics. However, 93% (*n* = 25) included studies in this review focused on male players.

The generalizability of findings on male players to female soccer remains uncertain. One factor contributing to this uncertainty is the developmental differences between sexes, such as the earlier average maturation in girls compared to boys (e.g., Malina et al. 2017). Additionally, there are notable differences between the TID systems for male and female players. For instance, despite rising participation rates among girls in recent years, the number of participants in organized soccer remains significantly lower than their male counterparts, resulting in a smaller overall talent pool (Emmonds et al. 2024; Valenti et al. 2021). One consequence is that many girls participate in mixed‐gender teams during childhood and early adolescence before transitioning to female teams (Andrew et al. 2024; McEwan et al. 2024). This structure creates challenges for practitioners when evaluating female players' talent within male‐dominated training groups where a reference group of same‐aged female peers may be lacking. Furthermore, in some countries (e.g., Germany), female players directly transition from under‐17 (U17) to U20 or even adult teams, whereas male players typically pass through U18/U19 and sometimes even U21 squads before reaching the adult level. In combination, such developmental and structural differences may not only affect the predictive validity of performance factors across developmental stages but also influence methodological decisions in designing prospective talent research.

Specifically, the criterion variables that are used to define future success and the prognostic periods that are utilized to evaluate them are common moderators of the predictive power of performance factors (Bergkamp et al. 2019). According to recent literature reviews by Emmonds et al. (2024) and Randell et al. (2021), the predictive validity of talent assessments in female soccer has only been investigated in three prospective studies. These studies offer initial evidence on the predictive validity of objectively assessed technical, physical, and physiological predictors in female players. Höner et al. (2019) demonstrated the 5‐year prognostic relevance (U12 to U17) of sprint, agility (CODS), dribbling, and ball control tests for the selection into a regional association and youth national team with moderate to large effect sizes within the German Football Association's (Deutscher Fußball‐Bund [DFB]) TID program. Sprint and dribbling tests further discriminated with a moderate effect size between future youth national team players and individuals who were selected for regional association squads. Based on a longitudinal design within the same TID program, Leyhr et al. (2020) demonstrated that sprint, agility (CODS), dribbling, ball control, and shooting tests had a predictive value for players' participation in the first adult division in Germany. Yet, the longitudinal development of performance factors did not significantly predict players' adult performance level. Lastly, Datson et al. (2020) analyzed the predictive validity of sprint, countermovement jump, and high‐intensity endurance performance tests of female players (12.7–15.3 years old) participating in English Elite Performance Camps. Only high‐intensity endurance capacity was found to predict players' participation in competitive international squads in the age groups U17 to U20.

The overall limited number of such prospective studies highlights the general need to further address the gender data gap in talent research (Curran et al. 2019). In this effort, it seems especially important to address the lack of prospective studies that adopt a multidimensional approach (i.e., incorporating a wide range of potential talent predictors; Emmonds et al. 2024). This has been shown to be particularly predictive in male youth soccer when combining objective motor tests with subjective coach ratings (Dugdale et al. 2020; Höner et al. 2021; Sieghartsleitner et al. 2019). However, the predictive validity of subjective coach ratings and consequently their contribution to a multidimensional approach in female youth soccer remain largely unexplored (Emmonds et al. 2024). Furthermore, the predictive validity of talent assessments is typically moderated by the selection levels considered (e.g., due to the ratio of players categorized as successful and nonsuccessful) as well as the length of the prognostic period (Bergkamp et al. 2019). Consequently, there is a particular need for studies examining the predictive validity of multidimensional approaches across various career stages in female youth soccer (i.e., incorporating different selection levels) and involving examinations regarding the highest performance level in adulthood.

## 2 The Present Study

To address these gaps, this prospective cohort study aimed to analyze the predictive validity of subjective coach ratings and objective motor tests for female youth soccer players' future success at different selection levels and prognostic periods. The research was conducted within the nationwide German TID program in which the girls participate in one practice session per week at one of the 350 nationwide DFB base camps (BCs; “competence centers”; Kelly et al. 2024) to supplement their promotion in home clubs. Girls' participation at one of the BCs within the TID program begins at U12 and concludes after U15. Players within the program participate in annual objective and subjective assessments allowing for the consideration of multidimensional talent predictors (Höner et al. 2021). The current study was designed to evaluate the predictive validity of players' test performances during their final year of the program (i.e., U15), which represents a critical developmental trajectory in their soccer careers. Players' performance levels achieved in the U17 age group (i.e., over a two‐year period)—their final year in youth soccer in Germany before transitioning to adult teams—along with their performance levels achieved in adulthood were used as criterion variables. More specifically, the research aimed to address the following two research objectives: *First*, examine the univariate predictive validity of each subjectively and objectively assessed performance factor concerning the different criterion variables (Objective 1). *Second*, analyze the multivariate predictive validity by evaluating if combining both assessments increases the predictive validity (Objective 2).

## 3 Method

### 3.1 Sample

The sample of this prospective cohort study consisted of *N* = 264 female U15 players (*M*_age_ = 14.0 years, *SD*_age_ = 0.9) participating in the DFB TID program. These girls are among the top 2% of female football players in their respective age groups across Germany. They train in mixed‐gender groups alongside their male counterparts, who are among the top 4% of players in their age group. Participants took part in subjective and objective assessments in the 2015/16 (birth cohort 2001), 2016/17 (2002), or 2017/18 seasons (2003). Players' legal guardian/next of kin provided written informed consent for the collection and scientific use of the data before entering the program. The first author's university's ethics department approved the use of the data for this study.

### 3.2 Measures

#### 3.2.1 Subjective Coach Ratings

The subjective assessments of the players were conducted by BC coaches holding at least a UEFA B coaching license. These ratings were implemented in the 2015/2016 season to facilitate a detailed and systematic evaluation of players' competencies in solving specific game situations (DFB 2022). Beyond talent identification, a key objective of the individual assessment is to guide practice design and inform other coaching decisions (e.g., differentiated learning goals; Bergmann et al. 2024; Ford and Coughlan 2020). Accordingly, the ratings contribute to optimized talent development by complementing the insights derived from isolated motor tests. To ensure uniformity in the evaluations, coaches were provided with a detailed manual that explained each aspect of performance being assessed (DFB 2022). In total, the coaches subjectively rated players' performance across 14 characteristics: three items were related to *kicking skills* (i.e., kicking the ball with the dominant and nondominant leg, heading; Note: This translation is based on the German term Stoßtechniken, which encompasses a range of techniques involving one ball contact. In collaboration with native speakers, we carefully searched for an appropriate English equivalent and ultimately chose “kicking skills” to reflect the inclusion of all techniques involving hitting the ball. For example, it also covers passing skills, which are not considered “striking” techniques in a narrower sense), one item was related to *endurance*, seven items were related to *tactical skills* (i.e., behavior in offensive and defensive situations before, during, and after ball‐related actions, as well as game intelligence), and three items were related to *psychosocial skills* (i.e., motivational, volitional, and social skills). Items related to kicking skills, endurance, and tactical skills were each evaluated by coaches with reference to players' age group using a four‐point rating scale: “below‐average BC level” (0); “average BC level” (1); “level of the extended squad for the regional association team” (2); or “level of the core team for the regional association team” (3). Regarding psychosocial skills, coaches were asked to evaluate their players by using the scale: “below average level” (0); “average level (1); “high level” (2); or “very high level” (3). Reliabilities in terms of internal consistency (Cronbach's *α*) for tactical skills (*α* = 0.92) and psychosocial skills (*α* = 0.90) were excellent, whereas kicking skills showed at least satisfying values (*α* = 0.79). These values widely corresponded with evaluations involving male players (Höner et al. 2021). Therefore, single scores for each of the three skills (i.e., kicking, tactical, and psychosocial) were computed for further analyses along with endurance which consisted of a single item.

#### 3.2.2 Objective Motor Diagnostic

To complement coaches' subjective evaluations with objective information, the results of a motor test battery assessing speed abilities and technical skills were also utilized (DFB 2022). In addition to supporting the identification of particularly talented players, the detailed individual test results are intended to foster talent development through personalized training recommendations (Höner et al. 2020). Furthermore, the objective and standardized nature of the test allows for monitoring the progression of talent predictors over time (Leyhr et al. 2018). The assessment consisted of five individual tests: *sprint* (i.e., time to complete a 20‐m linear sprint), *agility* in terms of change of direction speed (CODS; i.e., time to complete a slalom course without a ball), *dribbling* (i.e., time to complete a slalom course with a ball), *ball control* (i.e., time needed to play six passes alternately to two opposing locations with at least two ball contacts), and ball *juggling* (i.e., ability to juggle the ball alternately with the left and right foot through as many subsections of a figure‐eight course as possible without ground contact). The execution times of the sprint, agility (CODS), and dribbling tests were measured using light barriers (Brower TC Timing, Draper, USA). The execution time of the ball control test was measured with hand‐stopped chronographs. Each test was performed twice, and the best result was included for subsequent analyses. Players were given a recovery period of at least 60 s between each test trial. The motor tests demonstrated good psychometric properties in terms of reliability, factorial validity, and predictive validity, as confirmed in several samples of both male (e.g., Höner et al. 2017; Höner et al. 2015) and female elite youth soccer players (Höner et al. 2019; Leyhr et al. 2020).

### 3.3 Criterion Variables

Players' future success was operationalized through three typical career trajectories in female soccer. First, it was determined if participants who were tested in U15 made the official roster for a team competing in the German *Women's U17 Bundesliga* two seasons after data collection. This league was implemented in 2012 to optimize development pathways for the most talented female players in Germany. In the considered seasons (2017/18 to 2019/20), this league was divided into three regional conferences, serving as one possible final step before entering professional women's soccer. The second criterion variable considering the same two‐year prognostic period was selection for the German *U17 Youth National Team*, which represents the highest possible selection level a player in that age class can achieve. Third, players' future success in adulthood was defined by participation in the *Women's Bundesliga* (seasons 2017/2018 to 2023/2024), which represents the highest performance level in Germany. For all three selection levels, only players who made an official match appearance were classified as “selected”, while all others were categorized as “nonselected”. Across these three distinct selection levels within the German TID pathway, it was possible that players in the study sample meet more than one criterion. These three criterion variables also allow to evaluate the potential influence of the different selection levels and prognostic periods on the assessment's predictive validity. Table 1 presents the selection rates as well as the overlaps of players within the respective subsamples.

**TABLE 1:** Numbers of selected players, selection rates and the overlaps of the sub‐samples that fulfill the different criterion variables (*N* = 264).

Criterion	*n* (Selection rate)	Overlaps with other criterion variables
U17 Bundesliga	115 (43.6%)	U17 National team: *n* = 15 Women's Bundesliga: *n* = 14
U17 National team	17 (6.4%)	Women's Bundesliga: *n* = 9
Women's Bundesliga	16 (6.1%)	—

### 3.4 Statistical Analyses

Statistical analyses were performed utilizing IBM SPSS (version 28), with a significance level set at *α* = 0.05. To check the data requirements for subsequent analyses, it was examined whether potential confounders reported in previous research—namely, differences across included birth cohorts and relative age effects (Elferink‐Gemser et al. 2012; Leyhr et al. 2021)—influenced the predictive power. Specifically, separate two‐way ANOVAs for each performance factor and future selection level were used to test potential interaction effects with birth cohort (i.e., 2001–2003) and birth quarter. As no significant interaction effects were found for birth cohort (*F(2, 258)* ≤ 2.78, *p* ≥ 0.06) or relative age (*F(3, 256)* ≤ 2.51, *p* ≥ 0.06) at any of the future selection levels, these variables were not considered further as covariates. Furthermore, as statistical power is a critical concern in prospective talent research (Bergkamp et al. 2019), post hoc sensitivity analyses were conducted to account for the distribution of the total sample (*N* = 264) across the three criterion variables. The minimum detectable population effect size for differences between two groups (Cohen's *d*) with predefined parameters (*α* = 0.05, 1–*β* = 0.80, two‐tailed) was calculated using G*Power (version 3.1.9.7; Faul et al. 2009). The analyses had sufficient sensitivity to detect small to moderate effect sizes for the prediction of players' participation in the U17 Bundesliga (*d* ≥ 0.31) and moderate effect sizes for the criteria U17 Youth National Teams (*d* ≥ 0.71) and Women's Bundesliga (*d* ≥ 0.73).

To analyze the *univariate predictive validity* (Objective 1) of each subjectively and objectively assessed predictor, independent‐sample *t*‐tests were conducted to investigate mean differences between participants of different selection levels. These were classified by Cohen's *d* (computed as the mean difference divided by the pooled standard deviation) including the respective 95% confidence intervals and were presented irrespective of their significance, given the restricted sensitivity of statistical analyses.

The data requirements to investigate the multivariate predictive validity (Objective 2) were evaluated through bivariate correlations among predictors. Correlations ranged from *r* = 0.02 (*p* = 0.70) for sprint and ball control to *r* = 0.79 (*p* < 0.001) for subjectively rated kicking skills and tactical skills. Variance inflation factors (*VIF*s) across predictors were all below 4.17, demonstrating no serious multicollinearity (Akinwande et al. 2015). Therefore, a logistic regression approach was chosen similar to that used by Höner et al. (2021), who evaluated the same assessments in a male sample from the same TID program. Three logistic regression models for each of the three criterion variables were computed in which the binary criterion variable was predicted by subjectively (Model 1), objectively (Model 2), and a combination of subjectively and objectively assessed performance factors (Model 3). Specifically, the independent variables for Model 1 comprised the four subjectively rated performance factors, whereas Model 2 included the five objective motor tests. Model 3 included all those nine assessed performance factors. Overall model fit was assessed using the likelihood ratio chi‐squared test and Nagelkerke's *R* ^2^. Regression coefficients and the odds ratio coefficients *e* ^*b*^ (including their 95% confidence intervals) were calculated to assess the player's relative chances to achieve a respective selection level, depending on the considered predictors. To facilitate comparisons of the effect sizes across predictors, the odds ratio coefficients *e* ^*b*^ were additionally adjusted to the standard deviations of the respective predictor. Thus, the resulting (*e* ^*b*^)^*SD*^ represents the relative change of the likelihood of achieving a respective selection level by a one standard deviation increase within the considered predictor. Effect sizes for both the univariate and multivariate analyses were consistently reported in a positive direction, regardless of whether the variable was negatively (i.e., lower times indicate higher performance) or positively coded (i.e., more points indicate higher performance), that is, better test performance resulted in higher chances of selection.

## 4 Results

### 4.1 Univariate Predictive Validity of Subjective and Objective Assessments (Objective 1)

**FIGURE 1:**
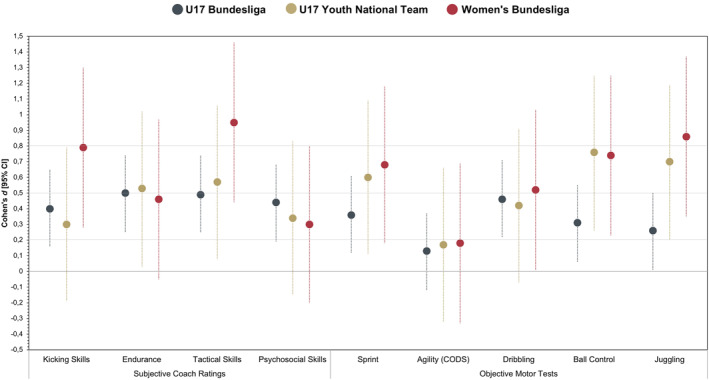
Superior performance in terms of Cohen's *d* and respective 95% CIs of future selected players among the three criterion variables.

The results of the univariate predictive validity for subjectively and objectively assessed predictors are displayed in Table 2 and Figure 1.

**TABLE 2:** Test results for subjectively (in italics) and objectively evaluated performance factors of U15 players (*N* = 264) based on the criterion variables of having participated in an official match in the U17 Bundesliga (*n* = 115; 43.6% selection rate), the U17 Youth National Team (*n* = 17; 6.4%), and the Women's Bundesliga (*n* = 16; 6.1%).

Performance factor	Future performance level	U17 Bundesliga		U17 Youth National Team		Women's Bundesliga	
		*M* ± *SD*	*t* (*df*)	*M* ± *SD*	*t* (*df*)	*M* ± *SD*	*t* (*df*)
			*Cohen's* *d*		*Cohen's* *d*		*Cohen's* *d*
			(95% CI)		(95% CI)		(95% CI)
*Kicking skills*	Selected	1.63 ± 0.56	3.25 (262) 0.40** (0.16; 0.65)	1.67 ± 0.49	1.20 (262) 0.30 (−0.19; 0.79)	1.92 ± 0.46	3.08 (262) 0.79** (0.28; 1.30)
	Nonselected	1.42 ± 0.53		1.50 ± 0.56		1.49 ± 0.55	
*Endurance*	Selected	2.20 ± 0.70	4.02 (262) 0.50*** (0.25; 0.74)	2.35 ± 0.61	2.11 (262) 0.53* (0.03; 1.02)	2.31 ± 0.79	1.80 (262) 0.46 (−0.04; 0.97)
	Nonselected	1.86 ± 0.67		1.98 ± 0.70		1.99 ± 0.69	
*Tactical skills*	Selected	1.90 ± 0.58	3.99 (262) 0.49*** (0.25; 0.74)	2.06 ± 0.53	2.28 (262) 0.57* (0.08; 1.06)	2.27 ± 0.54	3.71 (262) 0.95** (0.44; 1.46)
	Nonselected	1.61 ± 0.59		1.72 ± 0.60		1.70 ± 0.59	
*Psychosocial skills*	Selected	2.20 ± 0.64	3.52 (262) 0.44** (0.19; 0.68)	2.25 ± 0.66	1.36 (262) 0.34 (−0.15; 0.83)	2.23 ± 0.69	1.16 (262) 0.29 (−0.21; 0.80)
	Nonselected	1.92 ± 0.67		2.03 ± 0.67		2.03 ± 0.67	
Sprint (20 m)^#^	Selected	3.44 ± 0.17	2.92 (262) 0.36*** (0.12; 0.61)	3.39 ± 0.16	2.40 (262) 0.60* (0.11; 1.09)	3.38 ± 0.16	2.63 (262) 0.68** (0.17; 1.18)
	Nonselected	3.50 ± 0.15		3.48 ± 0.16		3.48 ± 0.16	
Agility (CODS)^#^	Selected	8.22 ± 0.37	1.01 (262) 0.13 (−0.12; 0.37)	8.19 ± 0.33	0.69 (262) 0.17 (−0.32; 0.66)	8.18 ± 0.37	0.70 (262) 0.18 (−0.32; 0.69)
	Nonselected	8.27 ± 0.36		8.25 ± 0.37		8.25 ± 0.36	
Dribbling^#^	Selected	10.76 ± 0.65	3.73 (262) 0.46*** (0.22; 0.71)	10.66 ± 0.71	1.69 (262) 0.42 (−0.07; 0.91)	10.60 ± 0.56	2.04 (262) 0.52* (0.02; 1.03)
	Nonselected	11.06 ± 0.65		10.94 ± 0.66		10.95 ± 0.67	
Ball control^#^	Selected	9.37 ± 1.25	2.48 (262) 0.31* (0.06; 0.55)	8.68 ± 0.89	3.03 (262) 0.76** (0.26; 1.25)	8.70 ± 0.87	2.89 (262) 0.74** (0.23; 1.25)
	Nonselected	9.76 ± 1.31		9.65 ± 1.30		9.65 ± 1.30	
Juggling	Selected	7.76 ± 5.98	2.03 (218.44) 0.26* (0.01; 0.50)	10.47 ± 7.05	2.15 (17.245) 0.70** (0.20 1.19)	11.31 ± 5.04	3.36 (262) 0.86** (0.35; 1.37)
	Nonselected	6.36 ± 4.93		6.72 ± 5.25		6.69 ± 5.36	

*Note:* ^#^These variables are negatively coded (i.e., a lower value represents a higher performance). A positive *d* value displays a better test result for the selected players regardless of whether the considered variable was negatively or positively coded.

*p < 0.05.

**p < 0.01.

***p < 0.001.

Regarding the *subjective coach ratings*, significant differences between future U17 Bundesliga players and the remaining sample were found for all predictors (each *p* < 0.01) with small to moderate effect sizes (*Mdn*(*d*) = 0.47; 0.40 ≤ *d* ≤ 0.50). For selection into the U17 Youth National Team, small to moderate effect sizes were observed (*Mdn*(*d*) = 0.44; 0.30 ≤ *d* ≤ 0.57) with significant differences in endurance (*p* < 0.001) and tactical skills (*p* < 0.001), but nonsignificant differences in kicking (*p* = 0.23) and psychosocial skills (*p* = 0.18). Future Women's Bundesliga players outperformed the remaining sample with small to large effect sizes (*Mdn*(*d*) = 0.63; 0.29 ≤ *d* ≤ 0.95). Although nonsignificant differences for endurance (*p* = 0.07) and psychosocial skills (*p* = 0.25) were found, significant differences for kicking skills and tactical skills (each *p* < 0.001) occurred. These ratings of kicking (*d* = 0.79) and tactical skills (*d* = 0.95) in predicting players' participation in the Women's Bundesliga yielded the highest effect sizes across all subjectively rated attributes and criterion variables, whereas the remaining subjective variables all had comparably small to moderate effect sizes (0.29 ≤ *d* ≤ 0.57; see Figure 1).

All *objective motor tests*, except for agility (CODS) (*p* = 0.31), significantly predicted players' participation in the U17 Bundesliga (each *p* < 0.05) with small effect sizes (*Mdn*(*d*) = 0.30; 0.13 ≤ *d* ≤ 0.46). For players' selection into the U17 Youth National Team, small to moderate effect sizes were observed (*Mdn*(*d*) = 0.60; 0.17 ≤ *d* ≤ 0.76) with significant differences in sprint (*p* < 0.05), ball control (*p* < 0.01), and juggling (*p* < 0.01). Regarding players' selection into a Women's Bundesliga team, effect sizes ranged from small to large (*Mdn*(*d*) = 0.68; 0.18 ≤ *d* ≤ 0.86) with significant differences among all motor tests (each *p* < 0.05), except for agility (CODS; *p* = 0.48). When comparing the relevance across the three criterion variables (see Figure 1), ball control and juggling revealed particular relevance for selection into the U17 Youth National Team and the Women's Bundesliga. Similarly, but to a lower extent, the predictive validity for the sprint test seemed to increase by selection levels from U17 Bundesliga to U17 Youth National Team and to the Women's Bundesliga. The agility (CODS) test did not predict any future performance level. Dribbling consistently showed moderate relevance to predict all future performance levels.

### 4.2 Multivariate Predictive Validity of the Multidimensional Assessment (Objective 2)

The overall fits of the subjective multivariate assessment model (Model 1) were significantly better compared to the null model in predicting players' participation in the U17 Bundesliga and the Women's Bundesliga (18.11 ≤ χ^2^(4) ≤ 18.48, each *p* < 0.001; 0.09 ≤ Nagelkerke's *R* ^2^ ≤ 0.18), but not for predicting selection to the U17 Youth National Team (χ^2^(4) ≤ 7.174, *p* = 0.13). The overall fits of the objective assessment models (Model 2) were significantly better compared to the null model regarding all three selection levels (18.30 ≤ χ^2^(4) ≤ 20.21, each *p* < 0.01; 0.11 ≤ Nagelkerke's *R* ^2^ ≤ 0.21). The explained variance across the three selection levels was slightly higher for the objective compared to the subjective assessment (i.e., U17 Bundesliga: 11% vs. 9%, U17 Youth National Team: 18% vs. 7%, Women's Bundesliga: 20% vs. 18%).

Hierarchical regressions further revealed that adding the subjective predictors (Model 1) to the objective predictors (Model 2) led to a significant increase in explained variance for predicting players' participation in the U17 Bundesliga (Δχ^2^(3) = 11.47, *p* < 0.01, ΔNagelkerke's *R* ^2^ *=* 0.05) and the Women's Bundesliga (Δχ^2^(3) = 11.48, *p* < 0.01, ΔNagelkerke's *R* ^2^ *=* 0.11). Consequently, the combined subjective and objective model (Model 3) demonstrated the highest predictive power for these selection levels. For predicting the U17 Youth National Team selection, the combined Model 3 did not led to a significant increase in the explained variance (Δχ^2^(3) = 3.11, *p* = 0.38, ΔNagelkerke's *R* ^2^ *=* 0.03), highlighting the objective assessment of Model 2 as the best‐fitting model. These final models are presented in Table 3 and reveal a trend toward a higher explained variance for higher selections levels (i.e., U17 Bundesliga: 16%, U17 Youth National Team: 18%, Women's Bundesliga: 31%).

**TABLE 3:** Logistic regression results for the prediction of players future success (final models) based on the criterion variables of having participated in an official match in the U17 Bundesliga (Model 3), U17 Youth National Team (Model 2), and Women's Bundesliga (Model 3).

Age group	Omnibus‐tests			Predictor	Logistic regression coefficients				(e^*b*^)^SD(#)^
	*χ* ^2^ (*df*)	*p*	Nagelkerke *R* ^2^		*b*	Wald	*p*	*e* ^*b*^ [95%‐CI]	
U17 Bundesliga	33.70 (9)	< 0.001	0.16	Constant	8.16	—	—	—	—
				**Sprint**	**−2.26**	**5.3**	**< 0.05**	**0.11 [0.02; 0.72]**	**1.43**
				Dribbling	−0.49	3.84	0.05	0.62 [0.38; 1.00]	—
				Ball control	−0.19	2.56	0.11	0.82 [0.65; 1.04]	—
				Agility (CODS)	0.61	2.09	0.15	1.84 [0,80; 4,21]	—
				*Endurance*	0.38	1.61	0.2	1.46 [0.81; 2.64]	—
				*Psychosocial skills*	0.25	0.7	0.4	1.28 [0.72; 2.27]	—
				Juggling	−0.01	0.1	0.75	0.99 [0.94; 1.05]	—
				*Tactical skills*	0.12	0.07	0.79	1.13 [0.47; 2.68]	—
				*Kicking skills*	0.07	0.03	0.87	1.07 [0.49; 2.33]	—
U17 Youth National Team	18.30 (5)	< 0.01	0.18	Constant	14.38	—	—	—	—
				**Ball control**	**−0.78**	**5.57**	**< 0.05**	**0.46 [0.24; 0.88]**	**2.74**
				**Sprint**	**−3.70**	**3.86**	**< 0.05**	**0.03 [0.00; 0.99]**	**1.80**
				Juggling	0.05	0.89	0.35	1.05 [0.95; 1.16]	—
				Agility (CODS)	0.39	0.23	0.64	1.48 [0.30; 7.36]	—
				Dribbling	−0.08	0.02	0.88	0.92 [0.33; 2.55]	
Women's Bundesliga	31.70 (9)	< 0.001	0.31	Constant	13.7	—	—	—	—
				***Tactical skills***	**2.39**	**5.12**	**< 0.05**	**10.92 [1.38; 86.57]**	**4.21**
				**Sprint**	**−4.49**	**4.86**	**< 0.05**	**0.01 [0.00; 0.61]**	**2.03**
				Ball control	−0.70	3.44	0.06	0.50 [0.24; 1.04]	—
				*Psychosocial skills*	−1.12	2.4	0.12	0.33 [0.08; 1.35]	—
				Juggling	0.05	0.83	0.36	1.05 [0.94; 1.18]	—
				*Endurance*	−0.44	0.48	0.49	0.64 [0.18; 2.25]	—
				Agility (CODS)	0.39	0.19	0.66	1.48 [0.26; 8.52]	—
				*Kicking skills*	0.29	0.12	0.73	1.34 [0.25; 7.15]	—
				Dribbling	−0.03	0.01	0.96	0.97 [0.29; 3.21]	—

*Note:* Predictors were ordered by increasing values with regard to the Wald statistic with predictors from subjective coach ratings in italics. Significant predictors are presented in bold font. ^(#)^In order to facilitate comparisons for effect sizes of individual predictors, the odds ratio coefficients *e* ^*b*^ were additionally adjusted to the standard deviations. The resulting (*e* ^*b*^)^*SD*^ represent the relative change of the likelihood for being selected by a one standard deviation increase within the considered predictor. For negatively coded predictors, the adjusted odds ratios were inverted and displayed as (*e* ^*b*^)^*−SD*^.

Table 3 further provides information on the significance of each predictor within these best‐fitting logistic regression models (details for all other models are presented in Supplements I–III). To predict players' participation in the U17 Bundesliga, only the sprint test reached significance (*p* < 0.05) and revealed (*e* ^*b*^)^*SD*^ = 1.43 times higher relative selection chances by a one standard deviation (*SD* = 0.16 s) increase in performance. In the objective model predicting the U17 Youth National Team selection, both ball control ((*e* ^*b*^)^*SD*^ = 2.74) and sprint ((*e* ^*b*^)^*SD*^ = 1.80) significantly contributed to the model (each *p* < 0.05). For the Women's Bundesliga criterion, both subjectively rated tactical skills ((*e* ^*b*^)^*SD*^ = 4.21) and sprint ((*e* ^*b*^)^*SD*^ = 2.03) were significant (each *p* < 0.05).

## 5 Discussion

This study is among the first prospective investigations of talent predictors in female soccer (Dugdale et al. 2020; Höner et al. 2021; Sieghartsleitner et al. 2019). The findings indicate the predictive validity of a multidimensional assessment used with a comparably large sample of U15 players. Specifically, the findings confirm that subjective coach ratings and objective motor tests have predictive power for players' future success over a two‐year period (i.e., to U17) as well as into adulthood (i.e., professional level). Nevertheless, differences in the predictive relevance across predictors were identified, and the multivariate evaluation showed limited incremental validity of the different performance factors. These findings should be discussed in light of the characteristics of the subjective and objective assessment methods (Höner et al. 2021), as well as potential moderating effects of the different criterion variables in terms of the selection levels and prognostic periods.

The three criterion variables showed varying success rates of selected players. The U17 Women's Bundesliga serves as a comparably less selective criterion, whereas the U17 Youth National Team and the Women's Bundesliga represent the highest levels of achievement. Here, evaluating players' participation in the U17 Women's Bundesliga is a novel criterion for TID research and the selection rate of 43% in this study is notably high in comparison to previous prospective talent research (Datson et al. 2020; Dugdale et al. 2021). This higher selection rate might be explained by the fact that to be selected for a DFB BC, all players in the sample were assumed to have sufficient talent to participate in training sessions with the top 4% of boys in the U15 age group (Kelly et al. 2024) and, therefore, possess comparably high‐performance capability. Selection into the U17 Youth National Team and the Women's Bundesliga represents the highest levels of achievement, reflecting much lower selection rates. It is noteworthy that Höner et al. (2019) previously reported a higher selection rate of 14.2% of U12 female players in the same TID program into the U17 Youth National Team. However, this might relate to the different birth cohorts, age groups (i.e., U12 instead of U15), and the resulting different prognostic periods (five years instead of two). The selection rate for future Women's Bundesliga players in this study (6.1%) aligns closely with findings from a longitudinal study on U12 to U15 female players (6.2%; Leyhr et al., 2020).

The relevance of individual predictors based on univariate group‐based comparisons (Objective 1) showed some discrepancies that warrant discussion considering the different assessment methods used. The subjectively assessed predictors consistently showed small to moderate effect sizes across variables for predicting future participation in the Women's Bundesliga, with only two notable exceptions: kicking skills (*d* = 0.79) and tactical skills (*d* = 0.95). Corresponding to the findings in male youth soccer (Höner et al. 2021), the results confirm that the subjectively rated performance factors reveal predictive validity also in female youth soccer. Furthermore, the relevance of subjectively rated endurance capacity corresponds to Datson et al. (2020), who identified the objectively assessed high‐intensity endurance capacity as a predictor for the selection into competitive U17‐U20 squads in England. It should be noted that the present study used a subjective single‐item rating to assess this predictor, whereas Datson et al. (2020) employed objective tests. Although objective endurance data provide more detailed insights, the subjective approach within the DFB TID program seems to offer a valuable complement for the identification and development of talent due to the efficiency and pragmatism of the assessment.

The predictive power of objectively assessed predictors demonstrated greater differences, ranging from negligible to large effect sizes. The sprint performance showed consistent relevance across all criterion variables with a trend toward greater effect sizes at higher selection levels. This consistent relevance aligns with previous findings highlighting linear sprinting performance as a critical predictor for future success (for reviews Murr, Feichtinger, et al. 2018; Williams et al. 2020). Agility (CODS), as another purely speed‐based test, showed nonsignificant and negligible effect sizes across all future performance levels, which appears to contradict findings by Höner et al. (2019) who reported moderate effects when comparing female youth national team players and nonselected players (*d* = 0.67). However, this apparent inconsistency may be explained by methodological differences between the two studies and, in particular, their respective study samples. That is, although Höner et al. (2019) investigated U12 players, the ongoing deselection processes for the U15 participants in the current sample may have led to a more homogeneous performance level. The dribbling test showed similar relevance across selection levels with slightly lower effect sizes compared to Höner et al. (2019) who identified dribbling as the most predictive test. The predictive power of the dribbling test is likely due to the fact that the test captures both speed abilities and technical skills (Höner et al. 2017), each of which has shown prognostic relevance when considered independently (Williams et al. 2020). Players' ball control performance showed considerable predictive power for the highly selective groups widely aligning with previous studies (*d* = 0.55; Höner et al. 2019; *d* = 0.50; Leyhr et al. 2020). Regarding juggling, the present study is the first that evaluated this technical skill test with female players and supported its predictive validity for all selection levels, but especially for the youth national team and the Women's Bundesliga. Overall, the consistent predictive power of the test battery coupled with the feasibility of its utilization highlights its value in identifying and developing female soccer talent.

The multivariate predictive validity of the assessments was also confirmed (Objective 2), with the best‐fitting models explaining considerable amounts of variance. Consequently, the study adds on previous research with male players that combining subjective and objective assessments can increase the predictive power (Höner et al. 2021; Sieghartsleitner et al. 2019). Nevertheless, the findings also highlight certain limitations of the assessments with only a limited number of predictors reaching significance in these models. Despite notable effect sizes, these results indicate restricted incremental validity and noticeable differences in the relevance of individual performance factors. Specifically, sprint performance significantly contributed to prediction across all selection levels (1.43 ≤ (*e* ^*b*^)^*SD*^ ≤ 2.03). To predict the selection for the U17 Youth National Team, ball control performance further contributed to Model 2 ((*e* ^*b*^)^*SD*^ = 2.74), but adding subjective predictors did not significantly improve the model (only) for this selection level. Regarding players' participation in the Women's Bundesliga, subjectively rated tactical skills significantly contributed to the prediction with the highest effect size across all predictors ((*e* ^*b*^)^*SD*^ = 4.21). Although this emphasizes generally high predictive power of coach ratings, this similarly suggests limited additional value from other variables. Although this issue was already noted in previous research with male players (Höner et al. 2021), it appears more pronounced in this female sample.

Subjective ratings can, in principle, offer valuable advantages, such as incorporating long‐term observations and in‐game behavior (Lath et al. 2021; Roberts et al. 2019). However, three specific factors may have affected the accuracy of the judgments in the present study. First, all players were part of an already high‐performing, preselected group. This may have amplified the “restriction of range of talent” issue (Bergkamp et al. 2019), making it harder to differentiate between players in this homogenous group. This echoes previous research showing subjective ratings as less sensitive compared to objective assessments, especially when aiming to discriminate between players at rather comparable performance levels (Dugdale et al. 2020). Second, the ratings can be further affected by reference group effects that can occur when people's characteristics are evaluated within salient reference groups (Rothenbusch et al. 2016; Schild et al. 2018). Girls in the present study were observed and rated within a high‐performing male‐dominated training group, which is not uncommon in female soccer TID (Andrew et al. 2024; McEwan et al. 2024). Yet, this seems particularly challenging for the coaches as players need to be rated with reference to individuals of same age and sex. As only few girls compete at each BC, lacking reference to other talented girls can challenge coaches in judging players accurately. Third, it is important to highlight that coaches at BCs who conducted the subjective ratings have commonly more experience in working with male youth players. Therefore, potential gender‐specific differences (e.g., different talent pathways, talent pool size, and maturational processes) may have led to less accurate ratings. Consequently, the findings underscore the necessity to further educate coaches, especially to facilitate their diagnostic competence in terms of more selective observations and distinct ratings of individual performance factors. Similarly, increasing awareness of (gender‐specific) biases and strategies to mitigate them could improve the predictive validity of subjective assessments. To complement such engagement within coach education initiatives, a critical practical implication may be to encourage BC coaches to actively become involved with female‐specific talent development measures (e.g., regional association tournaments), as this can both foster a deeper understanding of the players' actual reference group and promote more nuanced and engaged interaction with the female talent pathway.

## 6 Limitations

While providing valuable insights into the predictive validity of a multidimensional assessment in female soccer, this study is not without limitations. Given the highly selective sample of U17 Youth National Team and Women's Bundesliga players, the sensitivity of statistical analyses addressing these criterion variables only allowed for detecting moderate effect sizes. Additionally, it should be noted that the findings of prospective studies are always tied to the structures of the respective talent development system which influence potential talent pathways and, consequently, the selection of criterion variables. Although female talent pathways tend to be more diverse than those of boys (Andrew et al. 2024), the DFB talent development program—particularly the mixed‐gender promotion of highly talented girls alongside boys at BCs—may represent a unique feature that limits the generalizability of the findings to other contexts. Furthermore, controlling for different talent pathways in Germany next to the U17 Bundesliga (e.g., early participation in U20 female teams of professional clubs) was not possible. However, given the small number of players who did not participate in the U17 Bundesliga but made it to the youth national team (*n* = 2; 12%) or the Women's Bundesliga in adulthood (*n* = 2; 13%), this likely had a minimal impact on the findings. In addition, the Women's U17 Bundesliga organized in regional conferences has been further reformed since the season 2024/25 in terms of a championship for female teams in combination to participation in regional male soccer leagues. In addition, it was not possible to control for the playing position in U15 as the DFB TID program widely follows a position‐independent promotion (Kelly et al. 2024). Lastly, a potential influence of players' biological maturation on the test performance as indicated by the previous research (Datson et al. 2020) was not considered. That said, future talent research with female players, should investigate whether (a) the biological maturity status already influenced players’ selection into the program (i.e., the amount of bias in the study sample), (b) it has an impact on players' success in the future, and (c) it potentially moderates the predictive validity of talent assessments.

## 7 Conclusion

This study is within the first to demonstrate the predictive validity of subjective coach ratings and objective motor tests, offering a multidimensional approach to TID in U15 female soccer. The findings highlight the contribution of combining these assessments in practice and emphasize the relevance to considering different talent predictors. A trend toward more predictive power for higher selection levels was observed, suggesting a moderating effect of selection levels and prognostic periods over two years (i.e., U17) and into adulthood. Additionally, the study uncovered specific insights relevant to TID research in female players. For instance, although coach ratings were generally predictive, conducting distinct evaluations for specific predictors proved challenging. Therefore, to further enhance TID for female players, it is crucial to educate practitioners on these nuances. More broadly, future TID research should focus on the developmental contexts in which female players are promoted. As shown in this and further studies (Andrew et al. 2024; McEwan et al. 2024), many future professional players trained and competed in mixed‐gender settings. Hence, it is vital to investigate whether girls who trained in mixed‐gender environments differed in their likelihood of reaching the professional level compared to those who trained in female‐specific environments.

## Conflicts of Interest

The authors declare no conflicts of interest.

## Supporting information

Supporting Information S1

## Data Availability

All datasets used in this study will be provided upon reasonable request.
