# Supplementary material for: Multidimensional Performance Assessments in U15 Female Soccer: The Predictive Validity for Different Selection Levels in U17 and Success in Adulthood
Source: Eur J Sport Sci. 2025 Jun 26;25(7):e12335. doi: 10.1002/ejsc.12335 (PMC12199725; doi:10.1002/ejsc.12335)
Supplement: Supplementary file 1 — Supporting Information S1 [file EJSC-25-e12335-s001.docx]

**Supplement 1.**

Models 1 and 2 aiming to predict players’ participation in the U17 Bundesliga.

| Criterion Variable | Omnibus-Tests | | | Predictor | Logistic regression coefficients | | | | (*e^b^*)*^SD(#)^* |  |
| --- | --- | --- | --- | --- | --- | --- | --- | --- | --- | --- |
|  |  |  |  |  |  |  |  |  |  |  |
|  |  |  |  |  |  |  |  |  |  |  |
|  | χ^2^ *(df)* | *p* | Nagelkerke *R*^2^ |  | *b* | Wald | *p* | *e^b^* [95%-CI] |  |  |
| Subjective Assessment (Model 1) | 18.48 (4) | < .01 | 0.09 | Constant | -2,07 | - | - | - | - |  |
|  |  |  |  | *Endurance* | 0.38 | 1.77 | 0.18 | 1.46 [0.84; 2.56] | - |  |
|  |  |  |  | *Tactical Skills* | 0.38 | 0.83 | 0.36 | 1.46 [0.65; 3.31] | - |  |
|  |  |  |  | *Psychosocial Skills* | 0.15 | 0.3 | 0.58 | 1.17 [0.67; 2.02] | - |  |
|  |  |  |  | *Kicking Skills* | 0.04 | 0.01 | 0.92 | 1.04 [0.49; 2.18] | - |  |
| Objective Assessment (Model 2) | 22.24 (5) | < .001 | 0.11 | Constant | 12.23 | - | - | - | - |  |
|  |  |  |  | Dribbling | -0,62 | 6.49 | < .05 | 0.54 [0.34; 0.87] | 1.51 |  |
|  |  |  |  | Sprint | -2,16 | 5.21 | < .05 | 0.12 [0.02; 0.74] | 1.41 |  |
|  |  |  |  | Ball control | -0,18 | 2.29 | 0.13 | 0.84 [0.67; 1.05] | - |  |
|  |  |  |  | Agility (CODS) | 0.42 | 1.04 | 0.31 | 1.52 [0.68; 3.38] | - |  |
|  |  |  |  | Juggling | 0 | 0.01 | 0.93 | 1.00 [0.95; 1.06] | - |  |

**Supplement 2.**

Models 1 and 3 aiming to predict players’ participation in the U17Youth National Teams.

| Criterion Variable | Omnibus-Tests | | | Predictor | Logistic regression coefficients | | | | (*e^b^*)*^SD(#)^* |  |
| --- | --- | --- | --- | --- | --- | --- | --- | --- | --- | --- |
|  |  |  |  |  |  |  |  |  |  |  |
|  |  |  |  |  |  |  |  |  |  |  |
|  | χ^2^ *(df)* | *p* | Nagelkerke *R*^2^ |  | *b* | Wald | *p* | *e^b^* [95%-CI] |  |  |
| Subjective Assessment (Model 1) | 7.17 (4) | 0.13 | 0.07 | Constant | -4.49 | - | - | - | - |  |
|  |  |  |  | *Tactical Skills* | 1.31 | 2.54 | 0.11 | 3.72 [0.74; 18.66] | - |  |
|  |  |  |  | *Endurance* | 0.63 | 1.09 | 0.3 | 1.87 [0.58; 6.10] | - |  |
|  |  |  |  | *Kicking Skills* | -0,75 | 1.08 | 0.3 | 0.47 [0.12; 1.95] | - |  |
|  |  |  |  | *Psychosocial Skills* | -0.4 | 0.44 | 0.51 | 0.67 [0.21; 2.16] | - |  |
| Subjective & objective Assessment (model 3) | 21.42 (9) | < .01 | 0.21 | Constant | 11.2 | - | - | - | - |  |
|  |  |  |  | Ball control | -0,71 | 4.38 | < .05 | 0.49 [0.25; 0.96] | 2.52 |  |
|  |  |  |  | Sprint | -3,91 | 4.04 | < .05 | 0.02 [0.00; 0.91] | 1.86 |  |
|  |  |  |  | *Tactical skills* | 0.93 | 1.09 | 0.3 | 2.54 [0.44; 14.67] | - |  |
|  |  |  |  | *Kicking skills* | -0,81 | 0.97 | 0.32 | 0.45 [0.09; 2.22] | - |  |
|  |  |  |  | *Endurance* | 0.55 | 0.7 | 0.4 | 1.73 [0.48; 6.30] | - |  |
|  |  |  |  | Juggling | 0.04 | 0.63 | 0.43 | 1.04 [0.94; 1.15] | - |  |
|  |  |  |  | Agility (CODS) | 0.44 | 0.29 | 0.59 | 1.56 [0.31; 7.84] | - |  |
|  |  |  |  | *Psychosocial skills* | -0,26 | 0.17 | 0.68 | 0.77 [0.23; 2.63] | - |  |
|  |  |  |  | Dribbling | 0.09 | 0.03 | 0.87 | 1.09 [0.39; 3.03] | - |  |

**Supplement 3.**

Models 1 and 2 aiming to predict players’ participation in the Women’s Bundesliga.

| Criterion Variable | Omnibus-Tests | | | Predictor | Logistic regression coefficients | | | | (*e^b^*)*^SD(#)^* |  |
| --- | --- | --- | --- | --- | --- | --- | --- | --- | --- | --- |
|  |  |  |  |  |  |  |  |  |  |  |
|  |  |  |  |  |  |  |  |  |  |  |
|  | χ^2^ *(df)* | *p* | Nagelkerke *R*^2^ |  | *b* | Wald | *p* | *e^b^* [95%-CI] |  |  |
| Subjective Assessment (Model 1) | 18.11 (4) | < .01 | 0.18 | Constant | -5.43 | - | - | - | - |  |
|  |  |  |  | *Tactical Skills* | 2.67 | 7.77 | < .01 | 14.37 [2.21; 93.62] | 4.96 |  |
|  |  |  |  | *Psychosocial Skills* | -1.25 | 3.59 | 0.06 | 0.29 [0.08; 1.04] | - |  |
|  |  |  |  | *Kicking Skills* | 0.26 | 0.12 | 0.73 | 1.30 [0.29; 5.76] | - |  |
|  |  |  |  | *Endurance* | -0.2 | 0.12 | 0.73 | 0.82 [0.26; 2.60] | - |  |
| Objective Assessment (Model 2) | 20.21 (5) | < .01 | 0.2 | Constant | 14.7 | - | - | - | - |  |
|  |  |  |  | Sprint | -4.03 | 4.37 | < .05 | 0.02 [0.00; 0.78] | 1.89 |  |
|  |  |  |  | Ball control | -0.67 | 3.95 | < .05 | 0.51 [0.27; 0,99] | 2.38 |  |
|  |  |  |  | Juggling | 0.08 | 2.09 | 0.15 | 1.08 [0.97; 1.20] | - |  |
|  |  |  |  | Agility (CODS) | 0.58 | 0.46 | 0.5 | 1.79 [0.33; 9.63] | - |  |
|  |  |  |  | Dribbling | -0.28 | 0.25 | 0.62 | 0.76 [0.25; 2.27] | - |  |
